# Upregulating miR-181b promotes ferroptosis in osteoarthritic chondrocytes by inhibiting *SLC7A11*

**DOI:** 10.1186/s12891-023-07003-7

**Published:** 2023-11-07

**Authors:** Dexin Wang, Yu Fang, Liang Lin, Wensuo Long, Lei Wang, Liwei Yu, Huaiming Deng, Dan Wang

**Affiliations:** 1 Department of Orthopaedics, Haishu Branch, Ningbo First Hospital, Ningbo, 315153 China; 2 Department of Pharmacology, Medical College of Dalian University, Dalian, 116622 China

**Keywords:** Osteoarthritis, Chondrocytes, Ferroptosis, miR-181b-5p, SLC7A11

## Abstract

**Background:**

Osteoarthritis (OA) is a common disease with a complex pathology. This study aimed to investigate the correlation between the aberrant upregulation of miR-181b and ferroptosis in chondrocytes during the progression of OA.

**Methods:**

An OA cell model was constructed with erastin. Ferrostatin-1 (Fer), bioinformatics, and dual-luciferase activity reports were used to investigate the effect of miR-181b on OA. Finally, a rat model of OA was induced by monosodium iodoacetate to verify that miR-181b inhibits SLC7A11 gene expression and increases ferroptosis.

**Results:**

The results showed that Fer could effectively reverse the erastin-induced inhibition of human chondrocyte viability, increase the level of collagenous proteins in human chondrocytes, and inhibit oxidative stress and ferroptosis. MiR-181b is abnormally elevated in OA cell models. Transfection of a miR-181b inhibitor could increase the expression levels of the ferroptosis-related proteins solute carrier family 7 members 11 (SLC7A11) and glutathione peroxidase 4 (GPX4), thereby inhibiting the occurrence of ferroptosis in chondrocytes. In addition, hsa-miR-181b-5p and SLC7A11 have a targeted regulatory effect. Transfection of *SLC7A11* siRNA effectively abrogated the increase in chondrocyte viability induced by the miR-181 inhibitor and increased ferroptosis. Finally, miR-181b was shown to exacerbate OA disease progression by inhibiting *SLC7A11* gene expression and increasing ferroptosis in a rat OA model.

**Conclusions:**

Elevating miR-181b may mediate chondrocyte ferroptosis by targeting SLC7A11 in OA.

**Supplementary Information:**

The online version contains supplementary material available at 10.1186/s12891-023-07003-7.

## Background

Osteoarthritis (OA) is the leading cause of disability in older people worldwide, and an estimated 300 million suffer from OA [1]. Excessive mechanical load on the joint may lead to the destruction of articular cartilage [2]. In addition to mechanical damage, chondrocyte damage can also be attributed to different types of programmed cell death, including necrosis, apoptosis, and autophagic cell death [3–5].

During OA progression, chondrocyte death and extracellular matrix degradation were involved in OA pathogenesis, and chondrocytes are often shown as typical standard features of ferroptosis [6]. Ferroptosis is constitutively controlled by intracellular GPX4 and can be inhibited by iron chelators and lipophilic antioxidants [7, 8]. Expression of GPX4 in the OA cartilage from OA patients was significantly lower than undamaged cartilage [9]. Importantly, GPX4 downregulation could increase the sensitivity of chondrocytes to oxidative stress and improve OA-like changes in chondrocytes [10]. Therefore, ferroptosis may be involved in the progression of OA. The intercellular level of glutathione (GSH) is mainly affected by solute carrier family 7 member 11 (SLC7A11), which is an essential constituent of the amino acid antiporter system xc– [11]. Inhibition of SLC7A11 inhibits glutathione activity and the depletion of intracellular GSH, resulting in elevated levels of lipid peroxides and leading to ferroptosis [12]. Many studies have shown that the activity of SLC7A11 can be regulated by a variety of miRNAs [13–16].

Numerous studies have shown that miRNAs are involved in the pathogenesis of OA and are critical controllers of cartilage formation and OA development [17–19]. Previous research has confirmed that microRNA-181a-5p (miR-181a-5p) is a critical mediator in destroying lumbar facet joint cartilage [20]. The hsa-miR-181a-5p reduces oxidation resistance by controlling SECISBP2 in OA [21]. The miR-181c can promote the proliferation of synovial cells in OA [22]. However, the role of miR-181b in OA has not been revealed. This study hypothesises that the upregulation of miR-181b may promote the progression of OA by targeting the SLC7A11 gene and mediating ferroptosis in chondrocytes.

## Materials and methods

### Cell culture

Human chondrocytes were purchased from Applied Biological Materials Inc. (T0020, Richmond, BC, Canada). The cells were cultured in a Prigrow IV medium containing 10% fetal bovine serum, 1% L-glutamine, and a 1% penicillin/streptomycin (Richmond, BC, Canada) solution. The cells were placed in a constant temperature incubator at 37 °C with 5% CO_2_. The protein expression of the human chondrocyte markers Aggrecan and collagen II was identified by immunofluorescence, according to Li et al. [23] The miR-181b mimic, inhibitor (miR-181b in) and negative control (miR-NC) were all purchased from GenePharma Technology Company (Suzhou, China). Transfection experiments were performed using Lipofectamine® 2000 (Thermo Fisher Scientific, USA). Cells were collected 48 h after transfection, and RT‒qPCR was used to determine transfection efficiency.

### Cell viability assay

Human chondrocyte viability was detected using Cell Counting Kit 8 (CCK-8) assays (Dojindo, Japan). Chondrocytes were seeded into 96-well plates at a density of 4000 cells per well. After 24 h, the cells were treated with 0, 0.25, 0.5, 1, 5, 10, 20, 40 µg/ml erastin, 1 µM ferrostatin-1 (Fer, an inhibitor of ferroptosis) or an equal volume of DMSO for 48 h. Then, 10 µL of CCK-8 solution was added to each well and incubated at 37 °C for 2 h in the dark. The absorbance was measured at 450 nm using a microplate reader (Molecular Devices, USA).

### Oxidative stress indicator detection

Control or transfected human chondrocytes were inoculated into 96-well plates at a density of 2 × 10^4^ cells/well and treated with 1 µM erastin, 1 µM Fer or DMSO for 48 h. The levels of malondialdehyde (MDA) and GSH in the supernatant were analyzed using kits from Nanjing Jiancheng Institute of Bioengineering (Nanjing, China) according to the manufacturer’s instructions.

### ROS detection

Intracellular ROS levels were detected by flow cytometry. Erastin-treated or transfected chondrocytes were centrifuged at 2000 × g for 5 min, and the supernatant was discarded. The remaining cell mass was resuspended in a medium containing 5 µM DCFH-Da and incubated in the dark for 30 min. The cells were rinsed twice with PBS. Finally, the cells were resuspended in 500 µL of PBS. ROS levels in the cell suspensions were then detected by flow cytometry (Attune, Life, USA).

### Iron level detection

Iron levels in cells were detected using an iron colorimetric assay kit (BioVision, USA). All procedures were performed according to the reagent instructions.

### qPCR

After total RNA was extracted from the cells by the TRIzol method, the mRNA was reverse transcribed into cDNA using the 1st Strand cDNA Synthesis Kit gDNA (Novoprotein, Shanghai, China), and miRNA was reverse transcribed using the MiRcute Enhanced miRNA cDNA First Strand Synthesis Kit (Tiangen, Beijing, China). Then, the PCR program was run on a 7500 Fast Real-Time PCR System (Applied Biosystems, Thermo Fisher Scientific, USA). The relative expression of each target gene was calculated using the 2^−ΔΔCt^ method. The sequences of the primers are shown in Tables 1 and 2.

**Table 1:** The sequences of the primers for rat human

Name	primer
hsa-miR-181b Forward	5’- TTC ATT GCT GTC GGT GG-3’
U6 Forward	5’-CTC GCT TCG GCA GCA CAT-3’
U6 Reverse	5’- TTT GCG TGT CAT CCT TGC G -3’
SLC7A11 Forward	5’- TCC TGC TTT GGC TCC ATG AAC G-3’
SLC7A11 Reverse	5’-AGA GGA GTG TGC TTG CGG ACA T-3’
GAPDH Forward	5’- GTC TCC TCT GAC TTC AAC AGC G-3’
GAPDH Reverse	5’- ACC ACC CTG TTG CTG TAG CCA A-3’

**Table 2:** The sequences of the primers for rat

Name	primer
Rno-miR-181b Forward	5’- TTC ATT GCT GTC GGT GG-3’
Rno-miR-181b Reverse	5’-GAA CAT GTC TGC GTA TCT C-3’
U6 Forward	5’-ATC TCG GAA GCT AAG CA-3’
U6 Reverse	5’-GGT CTC CCA TCC AAG TAC T-3’
SLC7A11 Forward	5’-TGA ATG CCT TGT CTG CTT TG-3’
SLC7A11 Reverse	5’-GAA TTG CAG GGA ACT GTG GT-3’
GAPDH Forward	5’-AGA CAG CCG CAT CTT CTT GT-3’
GAPDH Reverse	5’-CGA TAC GGC CAA ATC CGT TC-3’

### Western blot analysis

Cells were collected and lysed with RIPA lysis buffer. Proteins were separated by SDS‒PAGE and then transferred to PVDF membranes. After being blocked with 5% nonfat milk, the membrane was incubated with primary antibodies [collagen II, MMP-13, aggrecan (ACAN), FTH1, TFR1, p53, GPX4, GAPDH (Abcam, Cambridge, UK), and SLC7A11 (ABclonal, Wuhan, China)] overnight at 4 °C and with secondary antibodies for 2 h at room temperature. The protein bands were photographed using the BioSpectrum Imaging System (UVP, USA). The optical density of the protein bands was quantified by ImageJ software.

### Immunofluorescence staining

Human chondrocytes were seeded into 24-well plates that were pre-placed with the coverslip. After different treatments, the cells were fixed with 4% paraformaldehyde for 15 min at room temperature. The cells were treated with 0.5% Triton X-100 for 10 min and blocked with 1% BSA for 30 min at room temperature. The chondrocytes were then incubated with SLC7A11 antibodies overnight at 4 °C. The next day, the cells were incubated with FITC-conjugated goat anti-rabbit secondary antibodies for 1 h in the dark. After 3 washes with PBS, the cells were incubated with DAPI for 10 min. Images were acquired with a fluorescence microscope (Leica, Germany).

### Luciferase reporter gene assay

The potential target binding site of the human SLC7A11 gene 3’UTR sequence and miR-181b-5p was predicted by ENCORI (https://rna.sysu.edu.cn/encori/rriPathways. php). The dual-luciferase reporter gene plasmid was constructed using the pIS0 plasmid, and pRL-TK was used as the internal reference plasmid. The amount of transfected nucleic acid per well was as follows: Luc-3’UTR plasmid: 200 ng; pRL-TK 50 ng; miR-181b mimic and mimic NC: 2.5 µL (final concentration 100 nM). The cells were cotransfected with wild-type or mutant reporter gene plasmids, internal control plasmids, and the miR-181b mimic or mimic NC. After 48 h, the cells were collected. After the cells were washed, 80 µL of 1× passive buffer was added to each well to lyse the cells, and the cells were shaken at room temperature for 20 min. The wild-type SLC7A11 amplification primer sequences were as follows: *Mlu*I- upstream primer-5’-CG ACG CGT TGA AAC AGA TTG TTC CCA TGA ATG TA-3’, and *Xba*I- downstream primer -TGC TCT AGA GGA TCA GAT TAC TTA AAA TTG. The size of the amplified product was 198 bp.

### Cell transfection

The SLC7A11 siRNA sequence was designed using online design software (http://sidirect2.rnai.jp/). Then, 100 pmol of the SLC7A11-siRNA oligonucleotide sequence was transfected into human chondrocytes using Lipofectamine RNAiMAX. Transfection efficiency was determined by Western blotting 48 h after transfection. The SLC7A11-siRNA primer sequences are shown in Table 3.

**Table 3:** The SLC7A11-siRNA primer sequences

Name	primer
Oligo1: Forward	5’- AAG ACA AAG CUC CAA AUA GUG-3’
Oligo1: Reverse	5’- CUA UUU GGA GCU UUG UCU UAU-3’
Oligo2: Forward	5’- AAC AAA GUU GAG GUA AAA CCA − 3’
Oligo2: Reverse	5’- GUU UUA CCU CAA CUU UGU UAC − 3
Oligo3: Forward	5’- UGC UAA UGA GAA AUU UCC CAG − 3’
Oligo3: Reverse	5’- GGG AAA UUU CUC AUU AGC AGU-3’

### Establishment of the rat OA model

The rat OA model was established as previously described [24]. The rat adenovirus shSLC7A11 was constructed (1 × 10^8^ TU/mL). AntagomiR-181b was purchased from Ribo Biological Company (Guangzhou, China). The right knees of 24 rats were shaved and disinfected with 70% alcohol after inhalation anesthesia with isoflurane, and the rats were divided into 4 groups: 1, sham group (n = 6); 2, monosodium iodoacetate (MIA) model group (n = 6): given 3 mg MIA for OA induction; 3, antagomiR-181b group (n = 6): on the first day after OA induction, 100 pmol antagomiR-181b was injected into the knee joint immediately by micro syringe, which was repeated every 7 days; 4, antagomiR-181b + sh SLC7A11 group (n = 6): on the first day after OA induction, 50 µL adenovirus shSLC7A11 was injected into the knee joint immediately by micro syringe, along with 100 pmol antagomiR-181b, and the same treatment was performed every 7 days. The sham group was injected with 50 µL saline. The experiment was ended after 28 days. At the end of the experiment, all experimental animals were killed with carbon dioxide. All methods were conducted in compliance with the ARRIVE guidelines. The animal protocols were approved by the Animal Ethics Committee of the Second Hospital of Haishu District, Ningbo City (Approval No. 2020KY904).

### Histological assessment

The knees in each group were excised and fixed in 10% formalin at 4 °C for 24 h. Decalcification was performed using saturated EDTA-Na_2_ at 4 °C. Complete decalcification was followed by dehydration and paraffin embedding. Cartilage and mineralized bone tissue were stained with Safranin O-fast green staining. In addition, immunohistochemical (IHC) staining was performed to detect the distribution and expression of aggrecan, MMP-13 and SLC7A11 proteins as previously described [25]. The IHC results were quantified by Image-Pro Plus 6.0 software.

### Statistical analysis

All data in this study are presented as mean ± standard deviation based on three replicates. Graphs were present using GraphPad Prism Software (version Prism 8, GraphPad Software, Inc.). The normality of the data was assessed using the Shapiro-Wilk test. For data that followed a normal distribution, differences were analyzed by one-way ANOVA followed by Tukey’s post hoc test. For data that did not follow a normal distribution, the Kruskal-Wallis test was used. A P-value less than 0.05 was considered statistically significant.

## Results

### Erastin induces ferroptosis in chondrocytes

**Fig. 1:**
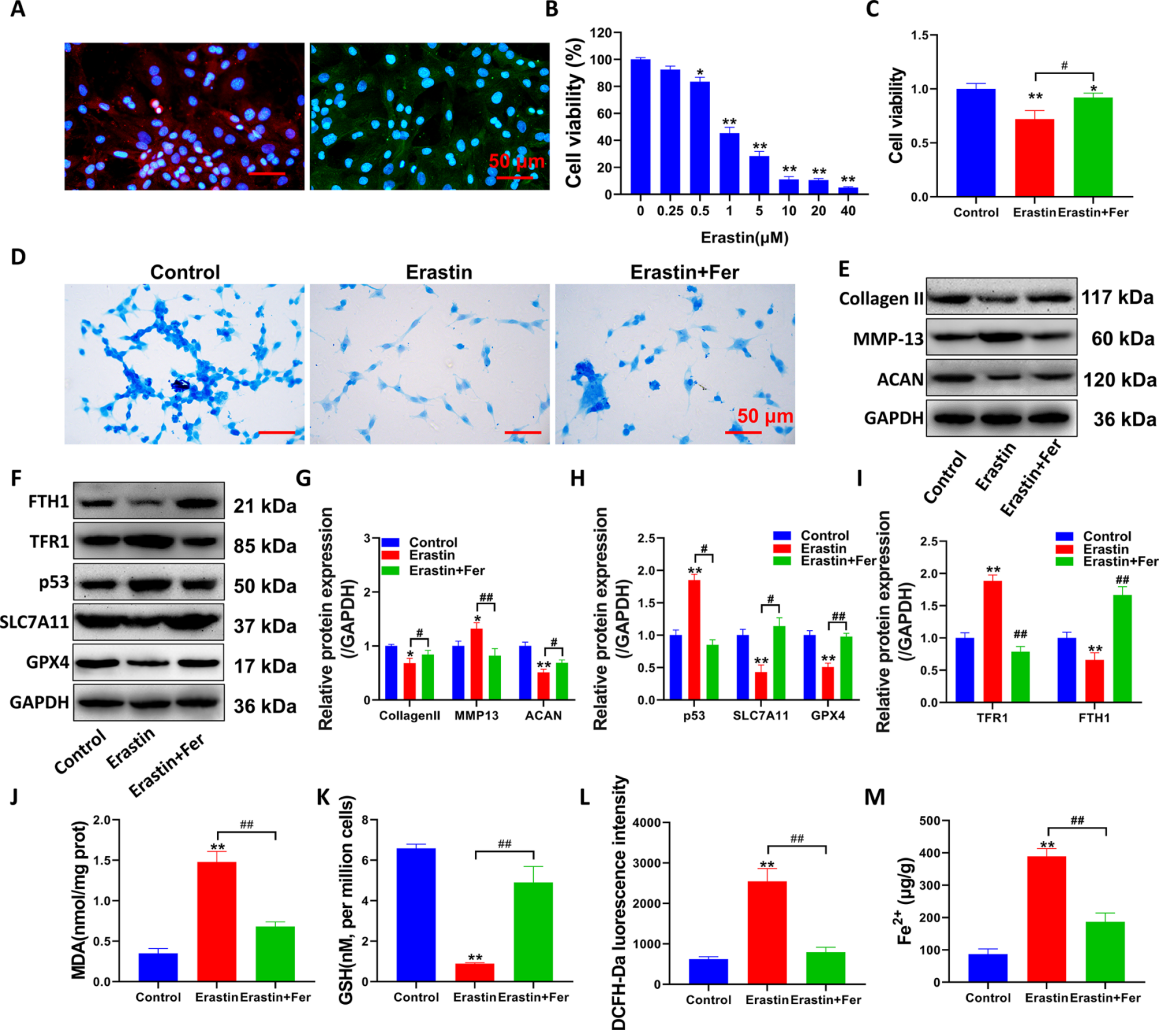
Erastin-induced ferroptosis in chondrocytes. (**A**) Immunofluorescence was used to detect the protein expression of aggrecan and collagen II. (**B-C**) CCK-8 detection of cell activity. (**D**) Alcian blue staining of chondrocyte matrix. (**E-F**) Western blot detection of matrix-related proteins (collagen II, ACNA and MMP13) and ferroptosis-related proteins (TFR1, FTH1, p53, SLC7A11, and GPX4) expression level. (**G-I**) Western blot optical density value quantification. (**J**) Cell MDA level detection. (**K**) Cell GSH level detection. (**L**) Flow cytometry analysis of cellular ROS accumulation and fluorescent labeling with DCFH-Da. (**M**) Detection of Fe^2+^ levels in cells. **P < 0.01, *P < 0.05, the difference between the model group and the control group was statistically significant. ##P < 0.01, #P < 0.05, the difference between the erastin + Fer group and model group was statistically significant

The results showed that the cells exhibited strong expression of aggrecan and collagen II proteins (Fig. 1A). Cell viability was significantly inhibited by at least 1 µM erastin (Fig. 1B, P < 0.01). The results showed that compared with the control group, erastin significantly inhibited cell viability after 48 h (P < 0.01, Fig. 1C). However, Fer significantly increased the viability of chondrocytes (P < 0.05, compared with the erastin group). Alcian blue staining demonstrated that after erastin treatment (Fig. 1D), the osteogenic extracellular matrix properties of chondrocytes were reduced, and inhibiting the occurrence of ferroptosis could reverse the inhibitory effect of erastin on chondrogenic properties. Erastin inhibited the expression of the cartilage extracellular matrix proteins collagen II and aggrecan and upregulated the expression of the matrix decomposition-related protein MMP-13 (Fig. 1E-I, P < 0.05, compared with the control group). Moreover, the expression of SLC7A11 and GPX4 was inhibited, and the expression level of p53 was upregulated. These above results indicated the occurrence of ferroptosis. (P < 0.05, compared with the control group). In contrast, the ferroptosis inhibitor Fer partially reversed the effect of erastin on chondrocyte production of collagen and ferroptosis-related proteins (P < 0.05, compared with the erastin group). TFR1 was significantly upregulated by erastin (P < 0.01 compared with the control group). The expression of FTH1 was significantly inhibited (compared with the control group, P < 0.01). These effects were reversed after Fer treatment (compared with the erastin group, P < 0.05).

Subsequently, indicators related to cellular oxidative stress were assessed following erastin treatment. Fer reversed the upregulation of MDA levels and the inhibition of GSH by erastin (Fig. 1J-K, P < 0.05, compared with the erastin group). Inhibiting ferroptosis significantly reduced the ROS accumulation in chondrocytes induced by erastin (Fig. 1L, P < 0.05, compared with the erastin group). After erastin treatment, the Fe^2+^ levels in chondrocytes increased significantly (Fig. 1M, P < 0.05, compared with the control group).

### Inhibition of miR-181b reverses erastin-mediated ferroptosis in chondrocytes

**Fig. 2:**
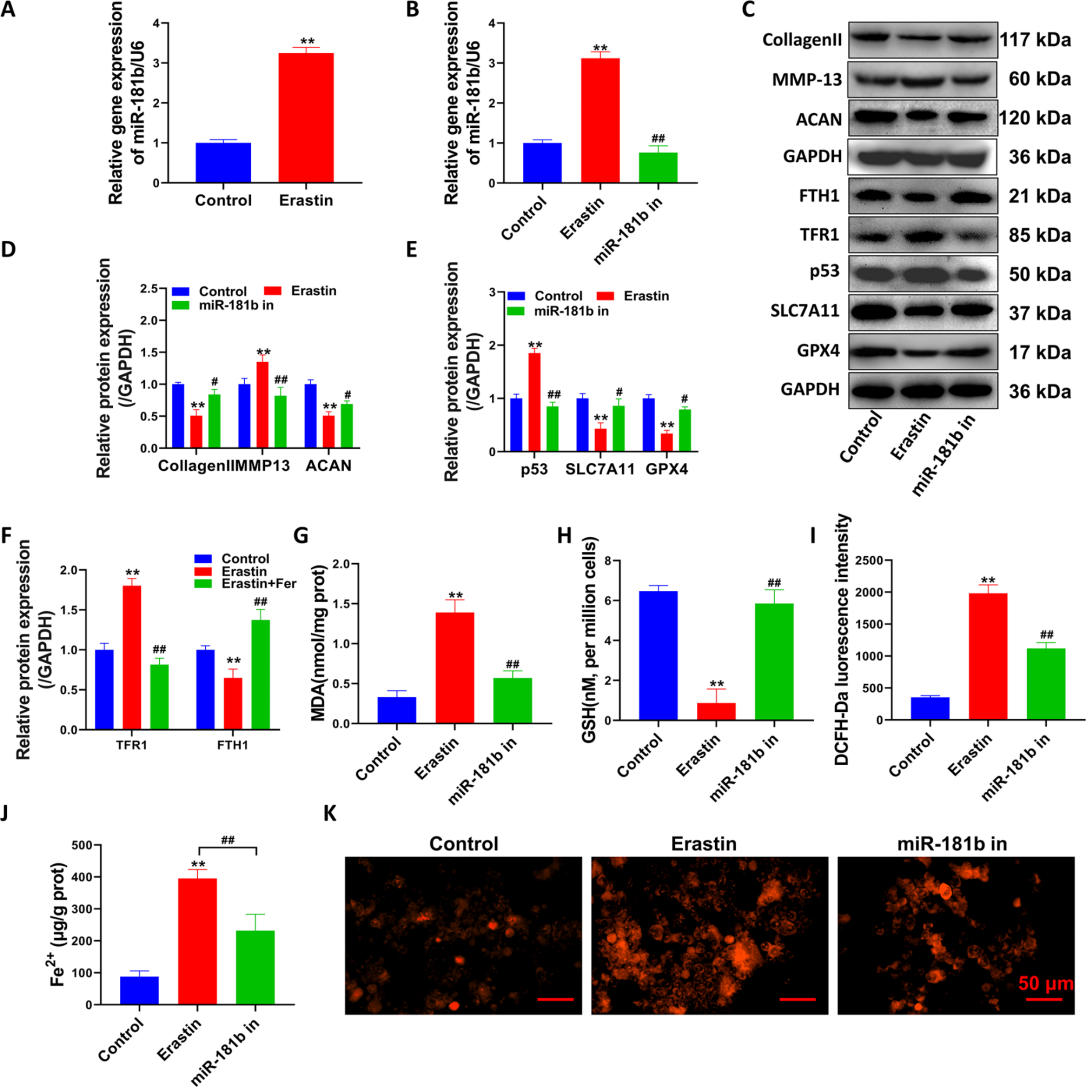
Inhibition of miR-181b inhibited the occurrence of ferroptosis. (**A-B**) qPCR detection of miR-181b gene expression levels. (**C**) Western blot detection of matrix-related protein and ferroptosis-related protein expression levels. (**D-F**) Western blot detection of bands and statistical quantification of the density values. (**G**) detection of MDA levels in cells. (**H**) Detection of GSH levels in cells. (**I**) ROS levels were detected by flow cytometry. (**J**) Detection of Fe^2+^ levels in cells. (**K**) Ferrous ions were stained with the Ferro Orange fluorescent probe. **P < 0.01, *P < 0.05, the difference between the model group and the control group was statistically significant. G-K. Erastin group: treated with 1 µM erastin, miR-181b group: treated with 1 µM erastin + miR-181b inhibitor. ##P < 0.01, #P < 0.05, the difference between the miR-181b in the group and the model group was statistically significant

Erastin treatment significantly upregulated the miR-181b gene in chondrocytes (P < 0.01, Fig. 2A). Figure 2B shows that the upregulation of the miR-181b gene by erastin was significantly inhibited after chondrocytes were transfected with the miR-181b inhibitor (P < 0.01). In the miR-181b inhibitor group, p53, MMP13, and TFR1 protein levels were significantly inhibited, while collagen II, ACNA, SLC7A11, GPX4, and FTH1 protein levels were significantly upregulated (Fig. 2C F, compared with the erastin group, P < 0.05). Inhibition of miR-181b significantly reduced the effect of erastin on the level of oxidative stress in chondrocytes (Fig. 2G-I, P < 0.01, compared with the erastin group). The accumulation of intracellular Fe^2+^ was significantly decreased after inhibiting miR-181b (Fig. 2J, P < 0.01, compared with the erastin group). The accumulation of ferrous ions in the cytoplasm was significantly enhanced after erastin treatment (Fig. 2K). Furthermore, the fluorescence intensity of miR-181b in the miR-181b inhibitor group was weakened, and only a few cells showed intense positive fluorescence probes.

### miR-181b regulates chondrocyte ferroptosis by targeting SLC7A11

**Fig. 3:**
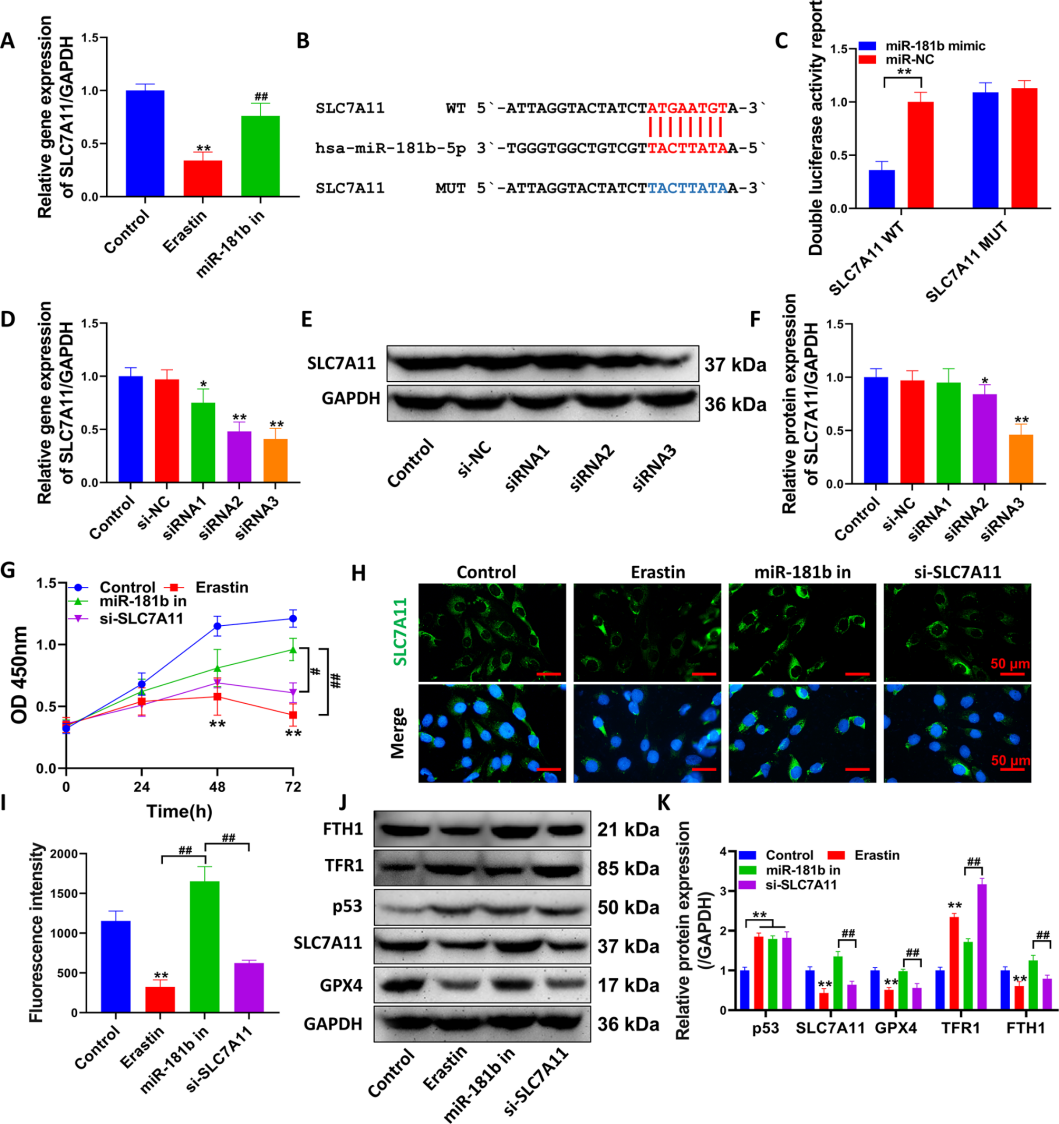
miR-181b mediates ferroptosis in chondrocytes by targeting SLC7A11. (**A**) qPCR detection of SLC7A11 gene expression. (**B**) hsa-miR-181b-5p and SLC7A11 target binding sequence and mutation sequence. (**C**) Dual luciferase activity reporter detection of the targeting relationship between hsa-miR-181b-5p and SLC7A11. (**D**) qPCR detection of SLC7A11 siRNA-mediated inhibition of SLC7A11 gene. (**E**) The inhibition efficiency of SLC7A11 siRNA on SLC7A11 protein expression was detected by Western blotting. (**F**) Statistical quantification of the optical density values of the Western blot bands. (**G**) CCK-8 assays to detect the effect of the miR-181b inhibitor or si-SLC7A11 on chondrocyte viability. (**H**) Immunofluorescence analysis to detect the expression level and distribution of SLC7A11. (**I**) Immunofluorescence analysis to quantify the fluorescence intensity. (**J-K**) Western blot analysis of the expression levels of ferroptosis-related proteins. **P < 0.01, *P < 0.05, compared with the control group, Erastin group: treated with 1 µM erastin, miR-181b group: treated with 1 µM erastin + miR-181b inhibitor, si-SLC7A11 group: treated with 1 µM erastin + si-SLC7A11. ##P < 0.01, #P < 0.05, the difference between the two groups is statistically significant

The qPCR results showed (Fig. 3A) that the SLC7A11 gene was significantly upregulated in chondrocytes transfected with the miR-181b inhibitor (P < 0.01, compared with the erastin group). To further confirm the regulatory relationship between these two factors, we constructed the SLC7A11 mutant gene sequence for the targeted binding site of miR-181b-5p and SLC7A11 (Fig. 3B). The dual-luciferase reporter assay demonstrated that miR-181b-5p had a targeted regulatory relationship with SLC7A11 (Fig. 3C). siRNA3 had the most potent inhibitory effect on the SLC7A11 gene and protein levels (Fig. 3D-F), and the difference was statistically significant compared with the control group (P < 0.01). In addition, siRNA3 reversed the inhibitory effect of erastin on chondrocyte viability after transfection with the miR-181b inhibitor (Fig. 3G, compared with the erastin group, P < 0.05). The effect of the miR-181b inhibitor alone on cell viability was attenuated by the cotransfection of SLC7A11 siRNA (P < 0.05, compared with the miR-181b inhibitor group in the si-SLC7A11 group). SLC7A11, GPX4 and FTH1 protein levels in the si-SLC7A11 group were significantly inhibited. In contrast, TFR1 protein expression was significantly upregulated, and the difference was statistically significant compared with the miR-181 inhibitor group (Fig. 3H-K, P < 0.05). However, there was no significant change in p53 protein levels after transfection with the miR-181b inhibitor or si-SLC7A11 (P > 0.05, compared with the erastin group).

### Effect of the miR-181b/SLC7A11 axis on MIA-induced osteoarticular cartilage injury in rats

**Fig. 4:**
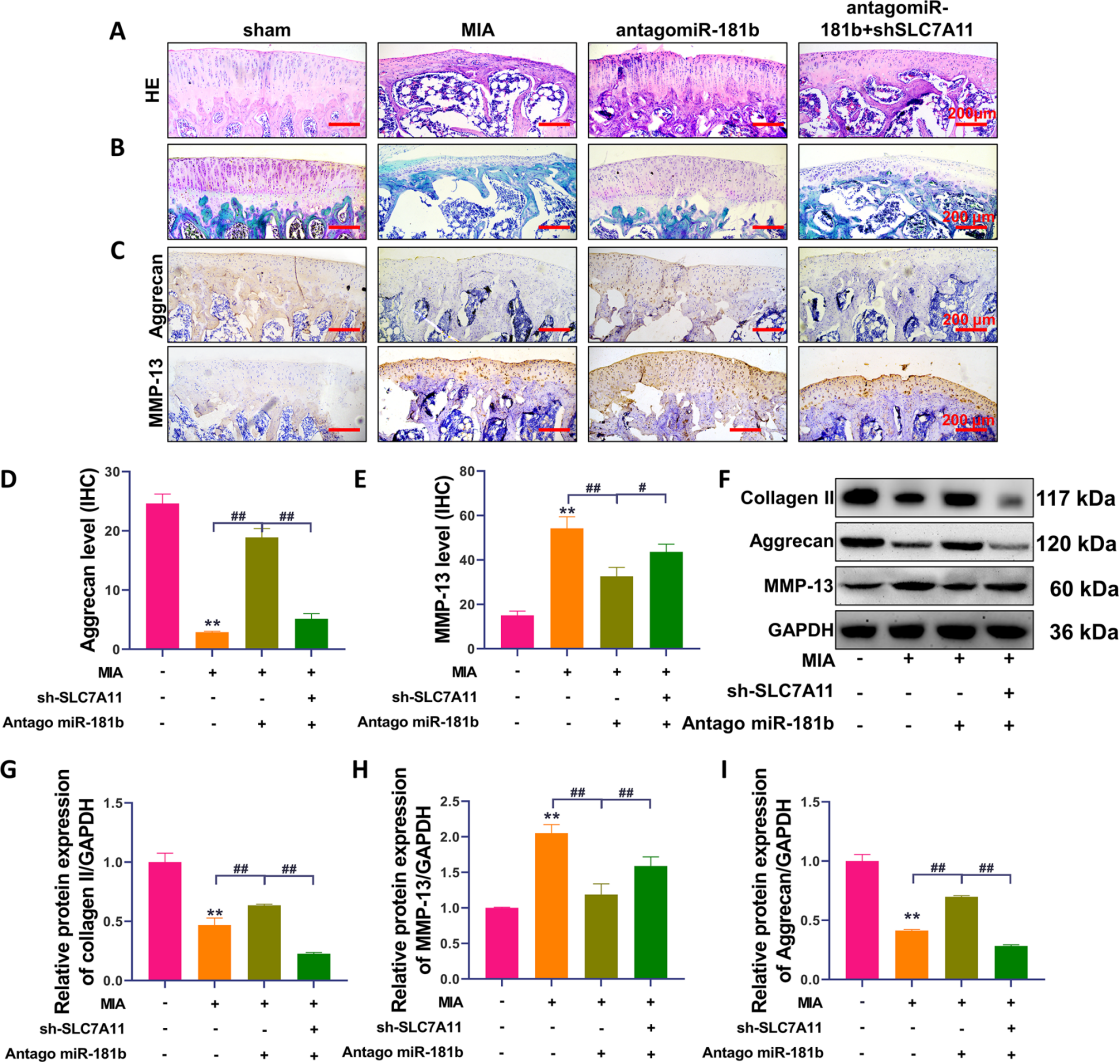
Effect of the miR-181b/SLC7A11 axis on MIA-induced osteoarticular cartilage injury in rats. (**A**) HE staining results. (**B**) Safranin O-fast green staining results. (C) IHC detection of aggrecan and MMP-13 protein expression levels. (**D-E**) Quantification of the IHC results. (**F-I**) Western blot analysis of collagen II, aggrecan and MMP-13 protein expression levels and the quantitative results. **P < 0.01, *P < 0.05, the model group was significantly different from the sham group. A-C: MIA group: treated with MIA, antagomiR-181b group: treated with MIA + antagomiR-181b, antagomiR-181b + sh-SLC7A11 group: treated with MIA + sh-SLC7A11. ##P < 0.01, #P < 0.05, the two groups of the line were compared, and the difference was statistically significant

HE staining of the OA model showed that inhibiting miR-181b expression significantly inhibited the MIA-induced reduction in the osteoarticular cartilage tissue layer and had a positive effect on the numerical recovery of trabecular bone (Fig. 4A). Silencing SLC7A11 significantly reversed the protective effect of antago-miR-181b on cartilage tissue. The distribution of glycan protein or the thickness of the cartilage tissue layer in the antago-miR-181b group was significantly better than that in the MIA group (Fig. 4B, red represents the cartilage tissue layer). IHC staining (Fig. 4C-E) and Western blotting (Fig. 4F-I) showed that collagen II and aggrecan protein expression was significantly enhanced, while MMP-13 expression was significantly inhibited in the antago-miR-181b group (P < 0.01, compared with the MIA group). However, the expression levels of these proteins were significantly reversed after sh SLC7A11-Ad injection (P < 0.05, compared with the antago-miR-181b group).

### The miR-181b/SLC7A11 axis regulates MIA-induced ferroptosis in rat cartilage tissues and joints

**Fig. 5:**
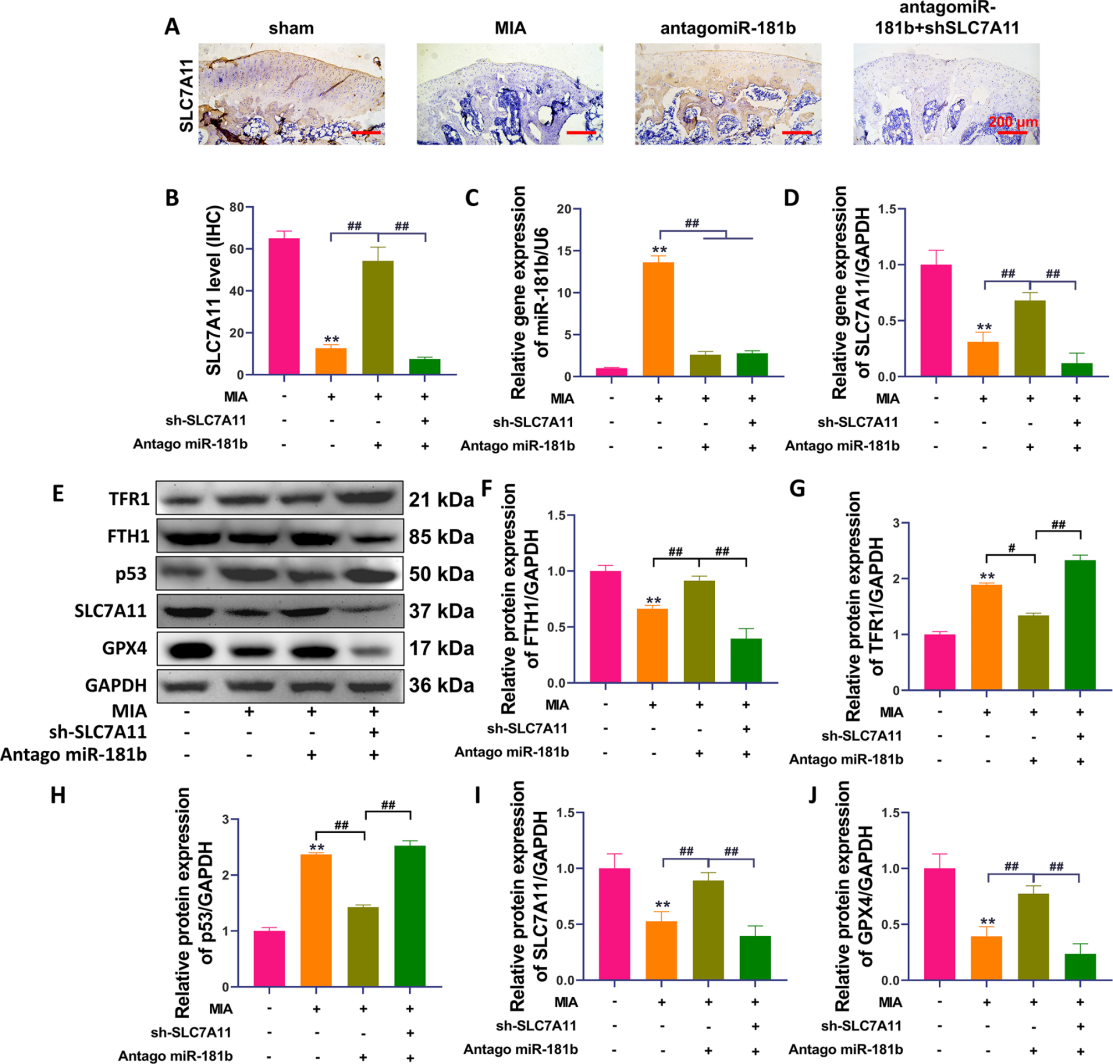
The miR-181b/SLC7A11 axis regulates MIA-induced rat osteoarticular ferroptosis. (**A-B**) IHC was used to detect the distribution and expression of SLC7A11 protein in bone tissue, and IPP6.0 was used for quantitative analysis. (**C-D**) qPCR was used to detect the expression levels of mir-181b and SLC7A11 genes. (**E-J**) The protein expression levels of TFR1, FTH1, p53, SLC7A11 and GPX4 in bone tissue were detected by Western blotting, and the optical density values of the protein bands were quantified. **P < 0.01, *P < 0.05, the model group was significantly different from the sham group. ##P < 0.01, #P < 0.05, the two groups of the line were compared, and the difference was statistically significant

**Fig. 6:**
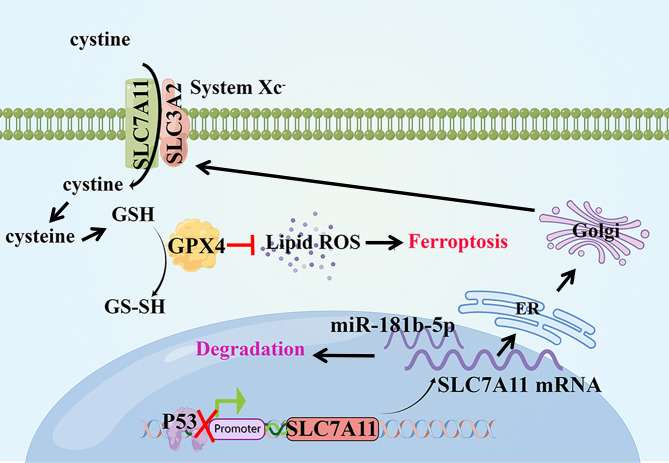
Diagram of the mechanism by which mir-181b-5p induces ferroptosis through SLC7A11. p53 represses SLC7A11 transcription by binding to the promoter area of SLC7A11. In addition, miR-181b-5p can inhibit the mRNA expression of SLC7A11 by binding to the SLC7A11 3’UTR. The important combinatorial protein SLC7A11 of System Xc^−^ can take extracellular cystine into the cell and convert it into reduced glutathione (GSH). GPX4 uses GSH as a substrate for lipid peroxidation repair and converts GSH into oxidized glutathione (GS-SH). Then, the occurrence of lipid peroxidation and ferroptosis is inhibited

The SLC7A11 protein was widely expressed in rat bone tissue under normal conditions, while SLC7A11 protein expression was significantly downregulated after MIA-induced OA in rats (Fig. 5A-B). Inhibiting miR-181b significantly upregulated SLC7A11 protein expression in cartilage tissue (P < 0.01, compared with the MIA group). The upregulation of SLC7A11 protein by antagomiR-181b was attenuated after silencing the SLC7A11 gene. AntagomiR-181b significantly inhibited miR-181b expression and upregulated the SLC7A11 gene in rat cartilage tissues (Fig. 5C-D, P < 0.01, compared with the MIA group). However, sh SLC7A11 significantly decreased the expression level of the SLC7A11 gene (P < 0.01, compared with the antagomiR-181b group). Finally, silencing SLC7A11 reversed the upregulation of the ferroptosis protection-related proteins FTH1, GPX4 and SLC7A11 in rat cartilage tissues in the presence of antagomiR-181b (Fig. 5E-J, P < 0.01, compared with the antagomiR-181b group). Figure 6 shows the mechanism by which the miR-181b gene promotes chondrocyte ferroptosis.

## Discussion

In recent years, ferroptosis in OA has gradually been recognized [3]. Yao et al. used chondrocytes extracted from C57BL/6J mouse knee joints as an in vitro experimental model of OA [26]. Zhou et al. found that D-mannose attenuated IL-1β-induced ferroptosis in chondrocytes, thereby exerting a chondroprotective effect [27]. In the erastin-induced OA cell model, Icariin reduces ferroptosis in joint synovial cells by activating the System Xc-/GPX4 axis and upregulating the expression of GPX4, SLC7A11, and SLC3A2L [28]. In the current study, we verified the importance of the miR-181b gene in erastin-induced ferroptosis in chondrocytes by using cell transfection technology. To further confirm the occurrence of erastin-induced ferroptosis in OA, the ferroptosis inhibitor Fer was also applied [26]. The rat OA model further verified that miR-181b promoted the development of OA through ferroptosis.

Members of the miR-181 family have been reported to play regulatory roles in controlling bone or chondrocyte development/function and mature chondrocyte homeostasis [20]. Inhibiting miR-181a-5p suppressed the expression of markers of inflammation, catabolism, and cell death and increased collagen II and MMP13 expression in OA chondrocytes [29]. Multiple studies have confirmed that the expression of miR-181b is upregulated in OA, and the decomposition of bone matrix is also increased [30]. This study showed the upregulation of miR-181b gene levels after erastin induction. Erastin increased cellular bone matrix degradation and ferroptosis-related proteins. This finding confirms that miR-181b may be necessary for chondrocyte ferroptosis induction.

It is worth noting that the p53 protein was not considerably regulated after transfection with the miR-181b inhibitor or SLC7A11 siRNA. p53 is a transcription factor that plays a crucial role in tumor suppression [31]. p53 binds to the p53 response element in the promoter region of SLC7A11 to inhibit its expression, thereby increasing the sensitivity of cells to ferroptosis-inducing agents such as erastin, and p53 deficiency causes the upregulation of SLC7A11, promoting ferroptosis resistance [32]. On the other hand, System Xc^−^ is a transmembrane cystine-glutamate antiporter that imports cystine into cells. GSH is a cofactor and substrate of GPX4 and is required for GPX4 to act as a key enzyme responsible for inhibiting lipid peroxidation. Depletion of GSH by cysteine starvation results in the loss of GPX4 activity, resulting in unrepaired lipid peroxide accumulation and ferroptosis [33]. Therefore, the inhibition of SLC7A11 may affect the activity of GPX4.

The regulation of miR-181b and SLC7A11 in this study only affected the protein levels of SLC7A11 and GPX4 but had no significant effect on the protein expression of p53 because the p53 gene is an upstream gene of SLC7A11. However, it is worth noting that miR-181b has shown different regulatory effects on p53 in various cancer studies [34].

## Conclusion

MiR-181b could inhibit ferroptosis by targeting the ferroptosis-protective protein SLC7A11. In addition, downregulating SLC7A11 reversed the inhibition of chondrocyte ferroptosis by antagomiR-181b, which in turn promoted OA progression. However, the cell model used in this study was constructed using erastin to create an OA cell model, and cell models may not fully reflect the real situation of human diseases. Therefore, further validation of these results in actual clinical samples is needed. In addition, the rat OA model used in the study was induced using sodium iodoacetate. Although animal models have important value in studying disease mechanisms, there may be differences between the rat model and the clinical manifestations and pathology of human OA.

### Electronic supplementary material

Below is the link to the electronic supplementary material.

Supplementary Material 1

## Data Availability

Data supporting the results of the survey can be obtained from the corresponding authors on request.
