# Supplementary material for: Upregulating miR-181b promotes ferroptosis in osteoarthritic chondrocytes by inhibiting SLC7A11
Source: BMC Musculoskelet Disord. 2023 Nov 7;24:862. doi: 10.1186/s12891-023-07003-7 (PMC10629093; doi:10.1186/s12891-023-07003-7)

Figure 1E

Collagen II
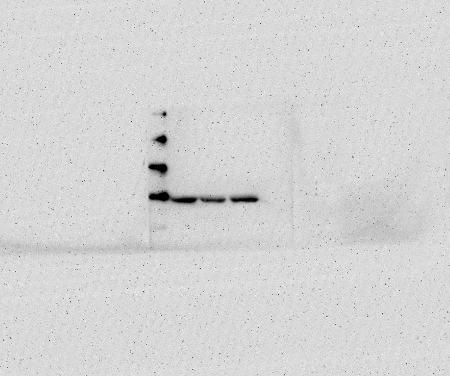
MMP-13
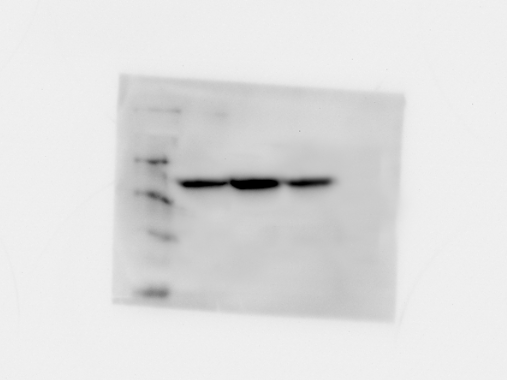


ACAN
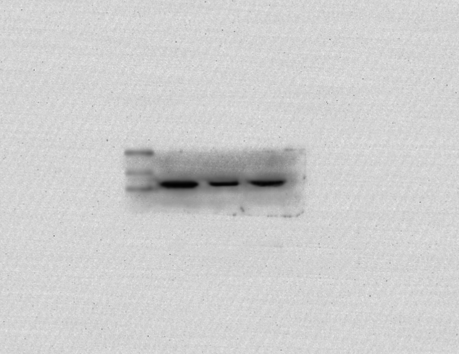
GAPDH
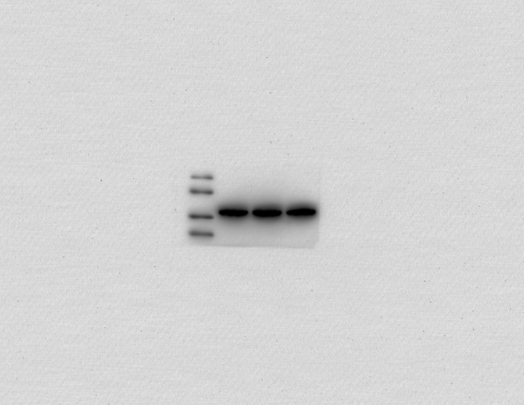


Figure 1F

FTH1
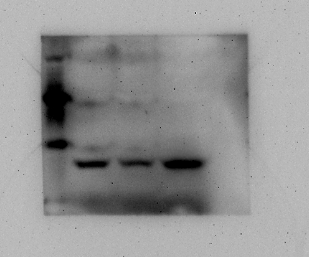
TFR1
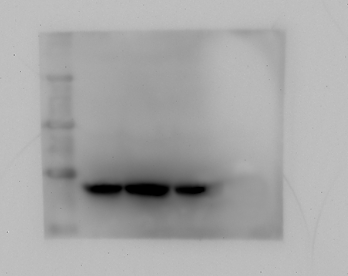


p53
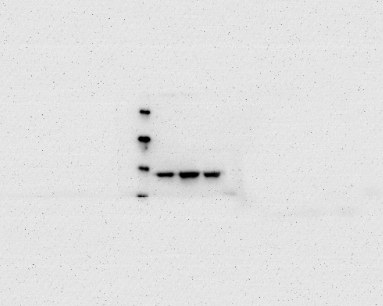
SLC7A11
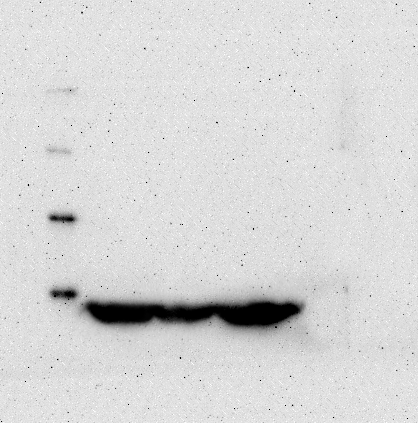


GPX4
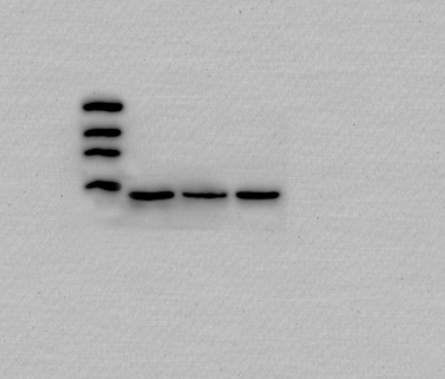
GAPDH
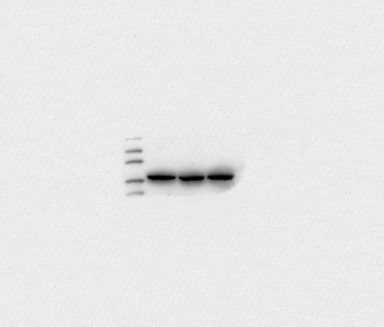


Figure 2C

CollagenII
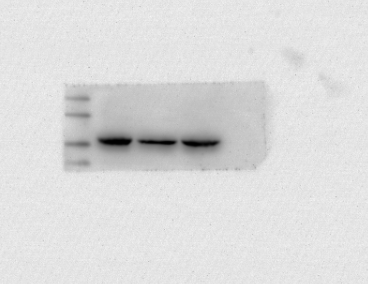
MMP-13
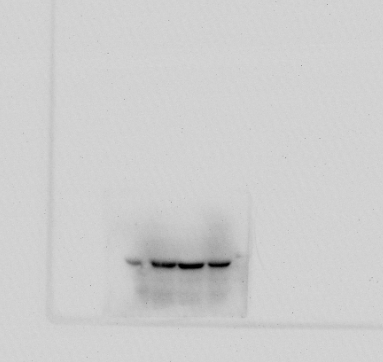


ACAN
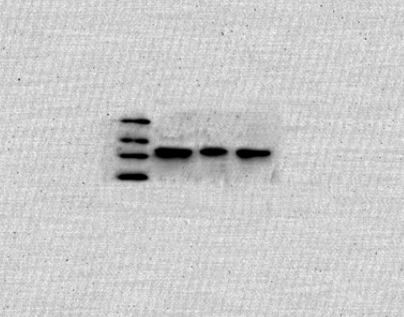
 GAPDH
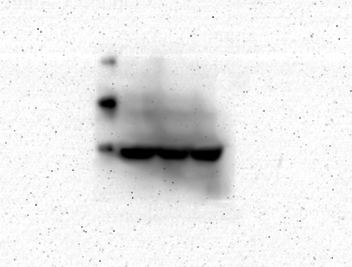


FTH1
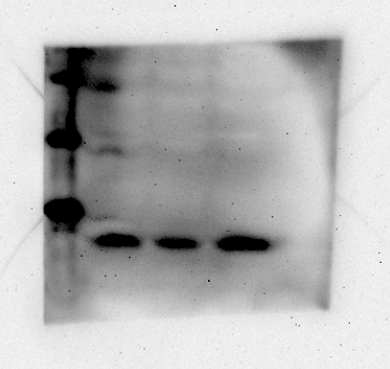
TFR1
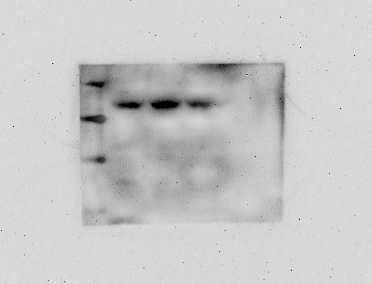


p53
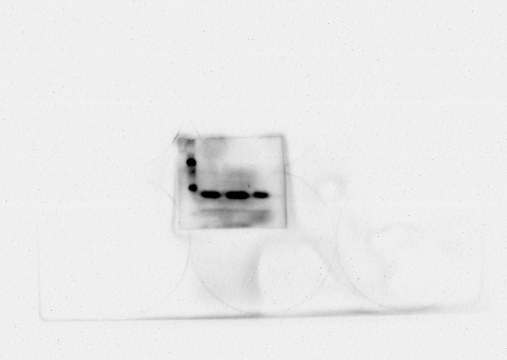
SLC7A11
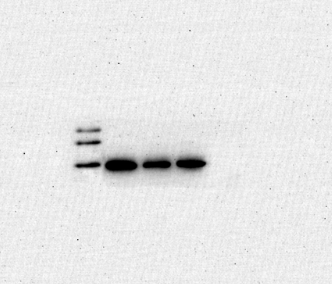


GPX4
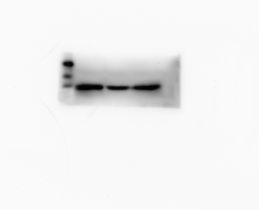
GAPDH
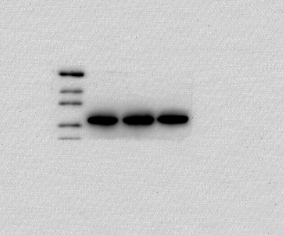


Figure 3E

SLC7A11
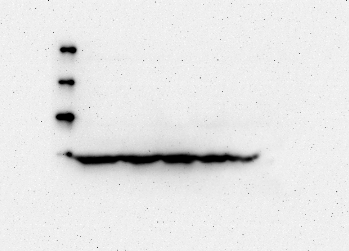
GAPDH
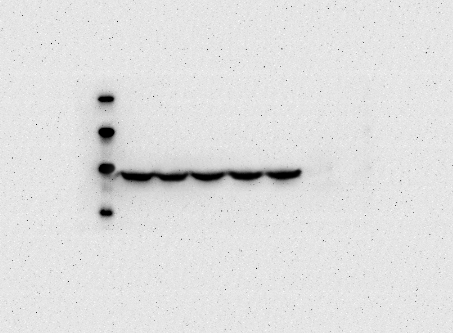


Figure 3J

FTH1
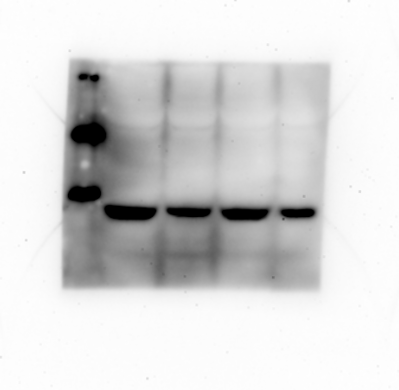
TFR1
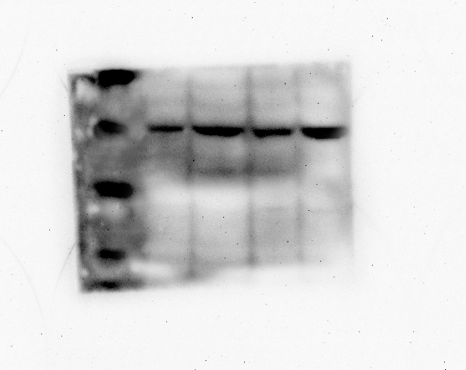


p53
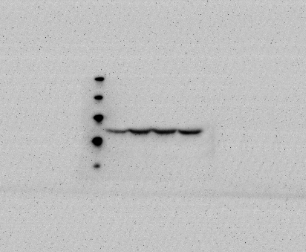
SLC7A11
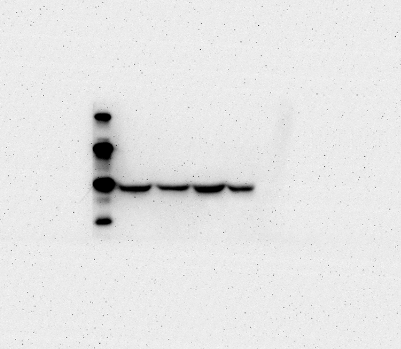


GPX4
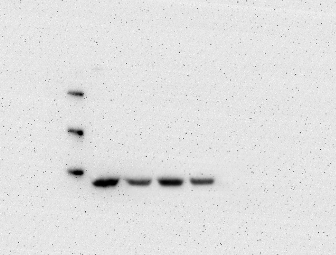
GAPDH
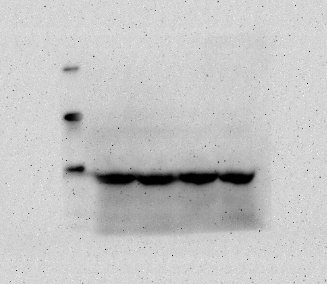


Figure 4F

Collagen II
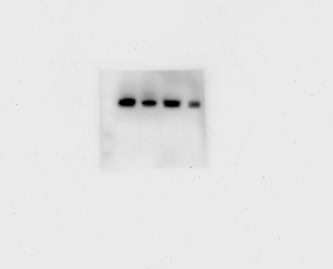
Aggrecan
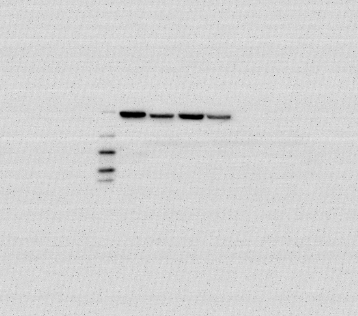


MMP-13
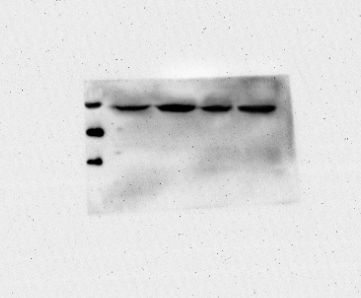
GAPDH
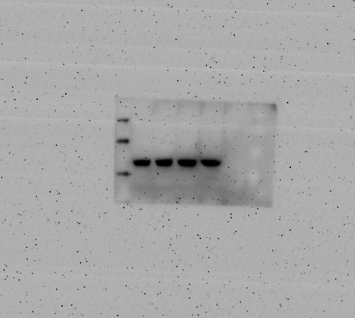


Figure5

TFR1
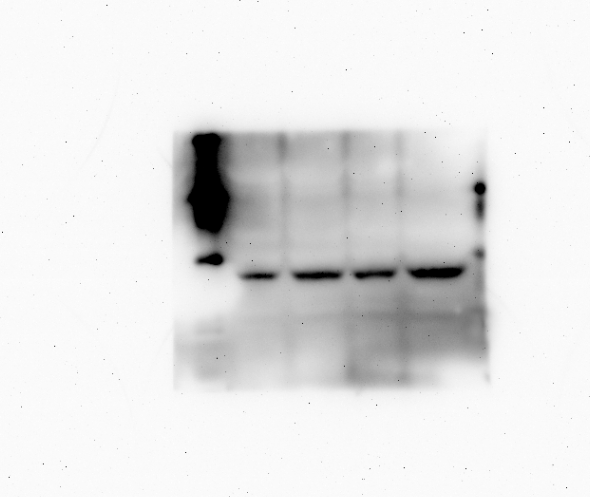
FTH1
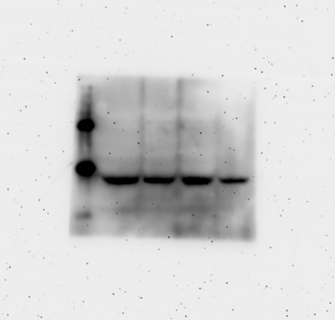


p53
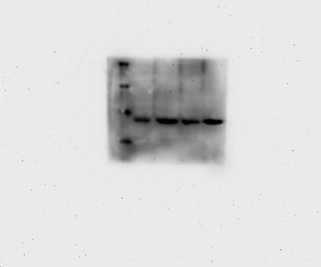
SLC7A11
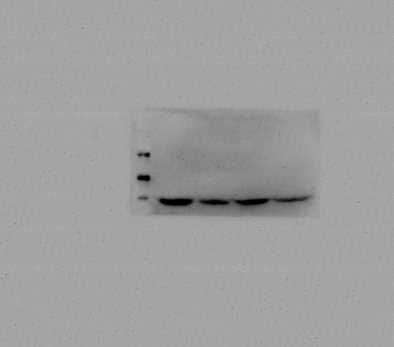


GPX4
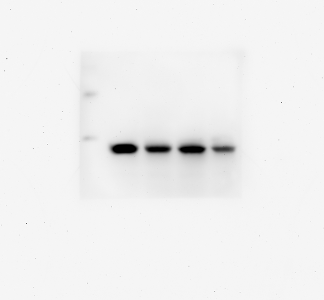
GAPDH
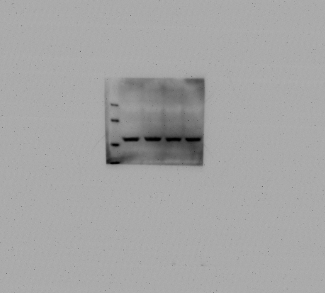

Supplement: Supplementary file 1 — Supplementary Material 1 [file 12891_2023_7003_MOESM1_ESM.doc]
